# 2,6-Bis(4*H*-1,2,4-triazol-4-yl)pyridine dihydrate

**DOI:** 10.1107/S1600536811022215

**Published:** 2011-06-11

**Authors:** Shang Yuan Liu, Ying Wang

**Affiliations:** a Tianjin Key Laboratory of Structure and Performance for Functional Molecules, Tianjin Normal University, Tianjin 300387, People’s Republic of China

## Abstract

In the asymmetric unit of the title compound, C_9_H_7_N_7_·2H_2_O, there are two formula units in which the two triazole rings of each of the organic component mol­ecules form dihedral angles of 7.0 (4)/6.9 (4) and 2.7 (4)/3.6 (4)° with the respective central pyridine rings. The four water mol­ecules of solvation form O—H⋯O hydrogen bonds among themselves and O—H⋯N bonds with the N-atom acceptors of the triazine rings, giving a three-dimensional framework structure.

## Related literature

For the synthesis of the title compound, see: Wiley & Hart (1953 ▶). For properties of related compounds, see: Haasnoot (2000 ▶).
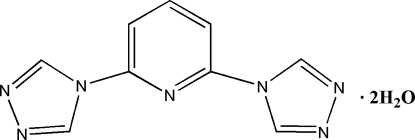


## Experimental

### 

#### Crystal data

C_9_H_7_N_7_·2H_2_O
*M* *_r_* = 249.25
Monoclinic, 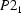

*a* = 7.052 (8) Å
*b* = 17.862 (16) Å
*c* = 9.715 (8) Å
β = 111.158 (9)°
*V* = 1141.2 (19) Å^3^
*Z* = 4
Mo *K*α radiation
μ = 0.11 mm^−1^
*T* = 293 K
0.30 × 0.29 × 0.10 mm

#### Data collection

Bruker APEXII CCD area-detector diffractometer
Absorption correction: multi-scan (*SADABS*; Sheldrick, 1996 ▶) *T*_min_ = 0.635, *T*_max_ = 1.000
6610 measured reflections
2096 independent reflections
1789 reflections with *I* > 2σ(*I*)
*R*_int_ = 0.028

#### Refinement

*R*[*F* ^2^ > 2σ(*F* ^2^)] = 0.036
*wR*(*F* ^2^) = 0.094
*S* = 1.19
2096 reflections
325 parameters
1 restraint
H-atom parameters constrained
Δρ_max_ = 0.13 e Å^−3^
Δρ_min_ = −0.19 e Å^−3^

### 

Data collection: *APEX2* (Bruker, 2008 ▶); cell refinement: *SAINT* (Bruker, 2008 ▶); data reduction: *SAINT*; program(s) used to solve structure: *SHELXS97* (Sheldrick, 2008 ▶); program(s) used to refine structure: *SHELXL97* (Sheldrick, 2008 ▶); molecular graphics: *SHELXTL* (Sheldrick, 2008 ▶); software used to prepare material for publication: *SHELXTL*.

## Supplementary Material

Crystal structure: contains datablock(s) I, global. DOI: 10.1107/S1600536811022215/zs2114sup1.cif

Structure factors: contains datablock(s) I. DOI: 10.1107/S1600536811022215/zs2114Isup2.hkl

Supplementary material file. DOI: 10.1107/S1600536811022215/zs2114Isup3.cml

Additional supplementary materials:  crystallographic information; 3D view; checkCIF report

## Figures and Tables

**Table 1:** Hydrogen-bond geometry (Å, °)

*D*—H⋯*A*	*D*—H	H⋯*A*	*D*⋯*A*	*D*—H⋯*A*
O1—H1*A*⋯N1^i^	0.85	1.98	2.814 (5)	165
O1—H1*B*⋯N7^ii^	0.85	2.07	2.904 (5)	168
O2—H2*A*⋯O4	0.85	1.87	2.709 (5)	169
O2—H2*B*⋯O1	0.85	2.00	2.850 (6)	174
O3—H3*A*⋯O1^iii^	0.85	2.05	2.888 (5)	169
O3—H3*B*⋯O2	0.85	2.07	2.916 (6)	170
O4—H4*A*⋯N13^iv^	0.85	1.98	2.822 (5)	170
O4—H4*B*⋯N8^v^	0.85	2.06	2.873 (5)	159

Symmetry codes: (i) 
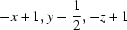
; (ii) 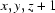; (iii) 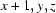; (iv) 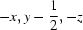; (v) 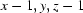.

**Fig. 1.**
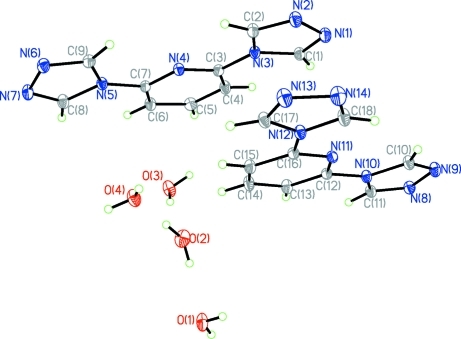
Molecular conformation and atom numbering scheme for the two independent molecules of C9H7N7 and the four water molecules of solvation in the asymmetric unit of (I). Probability spheres are drawn at the 15% level.

**Fig. 2.**
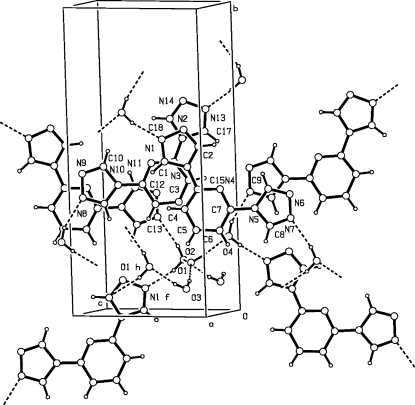
The packing of (I) in the unit cell showing hydrogen-bonding interactions.

## supplementary crystallographic information

### Comment

The field of molecular materials has known rapid development in recent years, with molecular-based compounds which exhibit interesting magnetic and luminescent properties having been described (Haasnoot, 2000). One of the requirements for producing such macroscopic properties is to create interactions between the molecular units and the active sites within the crystal lattices. 1,2,4-Triazole and in particular its derivatives are very interesting as bridging ligands. Here we report the synthesis and the crystal structure of the title compound, the dihydrate of 2,6-di(4*H*-1,2,4-triazol-4-yl)pyridine, C_9_H_7_N_7_ . 2(H_2_O) (I).

In the asymmetric unit of (I) there are two molecular units (Fig. 1) in which the two triazole rings of each of the organic component molecules form dihedral angles of 7.0, 6.9 (4)° and 2.7, 3.6 (4)° with the respective pyridine rings. The four water molecules of solvation form hydrogen bonds with the N atom acceptors only of the triazine rings (Table 1) giving a three-dimensional framework structure (Fig. 2).

### Experimental

A mixture of 1.3 g (0.012 mol) of 2,6-diaminopyridine and 2.0 g (0.023 mol) of diformylhydrazine was heated slowly to 160°C and held at 160-170°C for 30 min. The crystals, which separated on cooling, were collected and recrystallized from water to give 0.70 g of (I) (yield 13%). After several recrystallizations from water and from alcohol, the air-dried product was obtained as white needles, m.p. 325-327 K (placed in hot block at 320 K). The analysis was obtained on the air-dried sample. Anal. Calcd for C_9_H11_11_N_7_O_2_: C, 46.75; H, 3.92%. Found: C, 46.55; H, 3.96%.

### Refinement

Positional parameters of all the H atoms were calculated geometrically and were allowed to ride on the C or O atoms with C—H = 0.93 Å and O—H = 0.85 Å and *U*_iso_H = 1.2 or 1.5*U*_eq_(C or O). Friedel pairs were averaged for the data used in the final cycles of the refinement.

### Crystal data

**Table:**

C_9_H_7_N_7_·2H_2_O	*F*(000) = 520
*M**_r_* = 249.25	*D*_x_ = 1.451 Mg m^−^^3^
Monoclinic, *P*2_1_	Mo *K*α radiation, λ = 0.71073 Å
Hall symbol: P 2yb	Cell parameters from 1604 reflections
*a* = 7.052 (8) Å	θ = 2.3–23.1°
*b* = 17.862 (16) Å	µ = 0.11 mm^−^^1^
*c* = 9.715 (8) Å	*T* = 293 K
β = 111.158 (9)°	Block, colorless
*V* = 1141.2 (19) Å^3^	0.30 × 0.29 × 0.10 mm
*Z* = 4	

### Data collection

**Table:**

Bruker APEXII CCD area-detector diffractometer	2096 independent reflections
Radiation source: fine-focus sealed tube	1789 reflections with *I* > 2σ(*I*)
graphite	*R*_int_ = 0.028
φ and ω scans	θ_max_ = 25.0°, θ_min_ = 2.3°
Absorption correction: multi-scan (*SADABS*; Sheldrick, 1996)	*h* = −7→8
*T*_min_ = 0.635, *T*_max_ = 1.000	*k* = −20→21
6610 measured reflections	*l* = −11→9

### Refinement

**Table:**

Refinement on *F*^2^	Primary atom site location: structure-invariant direct methods
Least-squares matrix: full	Secondary atom site location: difference Fourier map
*R*[*F*^2^ > 2σ(*F*^2^)] = 0.036	Hydrogen site location: inferred from neighbouring sites
*wR*(*F*^2^) = 0.094	H-atom parameters constrained
*S* = 1.19	*w* = 1/[σ^2^(*F*_o_^2^) + (0.02*P*)^2^] where *P* = (*F*_o_^2^ + 2*F*_c_^2^)/3
2096 reflections	(Δ/σ)_max_ < 0.001
325 parameters	Δρ_max_ = 0.13 e Å^−^^3^
1 restraint	Δρ_min_ = −0.19 e Å^−^^3^

### Special details

**Table:**

Geometry. Bond distances, angles etc. have been calculated using the rounded fractional coordinates. All su's are estimated from the variances of the (full) variance-covariance matrix. The cell esds are taken into account in the estimation of distances, angles and torsion angles
Refinement. Refinement of *F*^2^ against ALL reflections. The weighted *R*-factor *wR* and goodness of fit *S* are based on *F*^2^, conventional *R*-factors *R* are based on *F*, with *F* set to zero for negative *F*^2^. The threshold expression of *F*^2^ > σ(*F*^2^) is used only for calculating *R*-factors(gt) *etc*. and is not relevant to the choice of reflections for refinement. *R*-factors based on *F*^2^ are statistically about twice as large as those based on *F*, and *R*- factors based on ALL data will be even larger.

### Fractional atomic coordinates and isotropic or equivalent isotropic displacement parameters (Å^2^)

**Table:**

	*x*	*y*	*z*	*U*_iso_*/*U*_eq_	
N1	0.8333 (4)	0.57130 (16)	0.3342 (3)	0.0478 (9)	
N2	0.7010 (5)	0.60054 (15)	0.2019 (3)	0.0486 (9)	
N3	0.7201 (4)	0.47959 (13)	0.1779 (2)	0.0362 (8)	
N4	0.5316 (4)	0.40426 (13)	−0.0145 (2)	0.0346 (8)	
N5	0.3281 (4)	0.33831 (14)	−0.2179 (2)	0.0361 (7)	
N6	0.1060 (4)	0.37967 (15)	−0.4259 (3)	0.0441 (9)	
N7	0.0990 (4)	0.30183 (16)	−0.4263 (3)	0.0481 (9)	
C1	0.8413 (5)	0.49967 (18)	0.3165 (3)	0.0397 (10)	
C2	0.6375 (5)	0.54465 (17)	0.1118 (3)	0.0438 (10)	
C3	0.6778 (4)	0.40638 (17)	0.1168 (3)	0.0349 (9)	
C4	0.7786 (5)	0.34433 (19)	0.1922 (3)	0.0439 (10)	
C5	0.7209 (5)	0.27547 (18)	0.1262 (3)	0.0454 (11)	
C6	0.5697 (5)	0.27085 (18)	−0.0111 (3)	0.0426 (10)	
C7	0.4817 (4)	0.33686 (17)	−0.0757 (3)	0.0342 (9)	
C8	0.2312 (5)	0.27945 (18)	−0.3025 (3)	0.0448 (11)	
C9	0.2422 (5)	0.39963 (17)	−0.3011 (3)	0.0400 (10)	
N8	0.9611 (4)	0.38269 (15)	0.9078 (3)	0.0438 (9)	
N9	0.9503 (4)	0.46049 (16)	0.9104 (3)	0.0476 (10)	
N10	0.7369 (4)	0.41986 (14)	0.6977 (2)	0.0359 (7)	
N11	0.5207 (4)	0.48637 (13)	0.5001 (3)	0.0349 (8)	
N12	0.3162 (4)	0.56230 (14)	0.3130 (2)	0.0373 (8)	
N13	0.1657 (5)	0.65496 (17)	0.1704 (3)	0.0554 (10)	
N14	0.2856 (5)	0.68389 (16)	0.3071 (3)	0.0569 (10)	
C10	0.8165 (5)	0.48092 (18)	0.7842 (3)	0.0451 (11)	
C11	0.8331 (5)	0.36112 (17)	0.7816 (3)	0.0399 (10)	
C12	0.5915 (4)	0.41913 (18)	0.5521 (3)	0.0343 (9)	
C13	0.5325 (5)	0.35351 (17)	0.4748 (3)	0.0436 (11)	
C14	0.3899 (5)	0.35832 (18)	0.3354 (3)	0.0477 (11)	
C15	0.3103 (5)	0.42704 (18)	0.2768 (3)	0.0426 (10)	
C16	0.3835 (4)	0.48866 (17)	0.3647 (3)	0.0339 (9)	
C17	0.1877 (5)	0.58319 (18)	0.1776 (3)	0.0450 (11)	
C18	0.3716 (5)	0.62737 (18)	0.3880 (3)	0.0507 (11)	
O1	0.0057 (4)	0.16140 (14)	0.4125 (2)	0.0636 (9)	
O2	0.3057 (5)	0.15947 (15)	0.2795 (3)	0.0725 (10)	
O3	0.6429 (5)	0.08242 (16)	0.2340 (3)	0.0749 (10)	
O4	0.1440 (4)	0.24252 (14)	0.0311 (2)	0.0625 (8)	
H1	0.91930	0.46660	0.38870	0.0480*	
H2	0.54740	0.54870	0.01480	0.0530*	
H4	0.88170	0.34870	0.28420	0.0530*	
H5	0.78400	0.23220	0.17450	0.0540*	
H6	0.52900	0.22510	−0.05800	0.0510*	
H8	0.25680	0.22950	−0.27490	0.0530*	
H9	0.27680	0.44890	−0.27220	0.0480*	
H10	0.78000	0.53030	0.75650	0.0540*	
H11	0.81000	0.31140	0.75220	0.0480*	
H13	0.58720	0.30770	0.51560	0.0520*	
H14	0.34610	0.31510	0.27950	0.0570*	
H15	0.21210	0.43130	0.18270	0.0510*	
H17	0.12400	0.55040	0.10040	0.0540*	
H18	0.46030	0.63090	0.48550	0.0610*	
H1A	0.04450	0.12750	0.47830	0.0760*	
H1B	0.02170	0.20560	0.44750	0.0760*	
H2A	0.26290	0.18190	0.19670	0.0870*	
H2B	0.22270	0.15830	0.32480	0.0870*	
H3A	0.75790	0.10000	0.28750	0.0900*	
H3B	0.53790	0.10490	0.23620	0.0900*	
H4A	0.06240	0.21210	−0.02880	0.0750*	
H4B	0.11000	0.28830	0.01600	0.0750*	

### Atomic displacement parameters (Å^2^)

**Table:**

	*U*^11^	*U*^22^	*U*^33^	*U*^12^	*U*^13^	*U*^23^
N1	0.0556 (17)	0.0435 (18)	0.0387 (14)	−0.0044 (14)	0.0102 (13)	−0.0062 (12)
N2	0.0578 (18)	0.0382 (16)	0.0420 (15)	−0.0014 (13)	0.0087 (13)	−0.0034 (12)
N3	0.0405 (14)	0.0313 (14)	0.0313 (12)	0.0007 (11)	0.0065 (11)	0.0000 (10)
N4	0.0365 (13)	0.0302 (15)	0.0320 (13)	0.0022 (10)	0.0061 (10)	0.0011 (10)
N5	0.0399 (13)	0.0300 (13)	0.0325 (12)	0.0003 (12)	0.0058 (10)	−0.0001 (11)
N6	0.0450 (15)	0.0418 (18)	0.0363 (15)	0.0030 (13)	0.0037 (13)	0.0064 (11)
N7	0.0549 (17)	0.0401 (18)	0.0361 (14)	−0.0026 (13)	0.0007 (13)	−0.0027 (11)
C1	0.0438 (17)	0.0359 (19)	0.0329 (16)	−0.0033 (15)	0.0060 (14)	−0.0015 (13)
C2	0.0513 (18)	0.0361 (19)	0.0365 (16)	−0.0009 (16)	0.0069 (14)	−0.0024 (14)
C3	0.0423 (17)	0.0285 (17)	0.0321 (15)	0.0009 (13)	0.0113 (13)	0.0007 (12)
C4	0.0451 (18)	0.0398 (18)	0.0367 (16)	0.0028 (15)	0.0026 (14)	0.0019 (14)
C5	0.0510 (19)	0.0327 (18)	0.0453 (18)	0.0074 (14)	0.0088 (15)	0.0118 (13)
C6	0.0530 (19)	0.0283 (17)	0.0399 (17)	0.0025 (14)	0.0090 (15)	0.0006 (12)
C7	0.0378 (16)	0.0309 (16)	0.0306 (14)	−0.0009 (14)	0.0085 (12)	0.0004 (13)
C8	0.053 (2)	0.0309 (18)	0.0411 (19)	−0.0016 (14)	0.0058 (16)	−0.0023 (13)
C9	0.0444 (17)	0.0328 (18)	0.0389 (17)	0.0037 (13)	0.0105 (14)	0.0047 (13)
N8	0.0491 (16)	0.0393 (17)	0.0369 (15)	0.0010 (12)	0.0082 (13)	0.0032 (11)
N9	0.0524 (17)	0.0416 (18)	0.0373 (15)	0.0004 (13)	0.0022 (13)	−0.0022 (12)
N10	0.0393 (13)	0.0306 (13)	0.0305 (12)	0.0007 (12)	0.0038 (10)	−0.0002 (11)
N11	0.0371 (14)	0.0328 (15)	0.0318 (12)	−0.0036 (11)	0.0088 (11)	−0.0003 (10)
N12	0.0427 (14)	0.0308 (15)	0.0307 (13)	0.0007 (11)	0.0039 (11)	0.0011 (10)
N13	0.0616 (18)	0.0407 (18)	0.0473 (16)	0.0017 (14)	−0.0004 (14)	0.0062 (12)
N14	0.070 (2)	0.0359 (16)	0.0506 (17)	0.0075 (14)	0.0045 (15)	0.0061 (13)
C10	0.051 (2)	0.0343 (19)	0.0425 (17)	−0.0004 (15)	0.0077 (16)	−0.0026 (14)
C11	0.0459 (17)	0.0308 (17)	0.0377 (16)	−0.0008 (13)	0.0087 (14)	0.0012 (12)
C12	0.0378 (15)	0.0316 (16)	0.0316 (14)	−0.0007 (13)	0.0102 (12)	0.0014 (13)
C13	0.054 (2)	0.0283 (18)	0.0412 (17)	0.0043 (14)	0.0083 (15)	−0.0008 (13)
C14	0.058 (2)	0.035 (2)	0.0407 (17)	−0.0034 (15)	0.0066 (16)	−0.0083 (14)
C15	0.0457 (18)	0.0387 (18)	0.0355 (15)	−0.0011 (15)	0.0050 (14)	−0.0013 (14)
C16	0.0363 (16)	0.0315 (17)	0.0316 (14)	−0.0002 (13)	0.0096 (13)	0.0021 (12)
C17	0.050 (2)	0.0362 (19)	0.0370 (17)	−0.0035 (15)	0.0014 (14)	0.0027 (14)
C18	0.064 (2)	0.0342 (19)	0.0401 (16)	0.0020 (17)	0.0021 (15)	−0.0022 (14)
O1	0.0880 (18)	0.0428 (14)	0.0407 (12)	−0.0062 (13)	0.0001 (12)	−0.0008 (10)
O2	0.0855 (19)	0.0621 (18)	0.0589 (15)	0.0193 (15)	0.0128 (14)	0.0157 (13)
O3	0.0718 (18)	0.0639 (17)	0.0754 (18)	−0.0105 (14)	0.0103 (14)	−0.0183 (14)
O4	0.0628 (15)	0.0468 (15)	0.0571 (13)	−0.0005 (12)	−0.0034 (12)	0.0059 (11)

### Geometric parameters (Å, °)

**Table:**

O1—H1A	0.8500	N11—C12	1.329 (4)
O1—H1B	0.8500	N12—C18	1.352 (4)
O2—H2B	0.8500	N12—C16	1.427 (4)
O2—H2A	0.8500	N12—C17	1.353 (4)
O3—H3A	0.8500	N13—C17	1.290 (5)
O3—H3B	0.8500	N13—N14	1.391 (4)
N1—N2	1.389 (4)	N14—C18	1.289 (4)
N1—C1	1.295 (5)	C3—C4	1.376 (5)
N2—C2	1.296 (4)	C4—C5	1.379 (5)
N3—C1	1.357 (4)	C5—C6	1.377 (4)
N3—C3	1.423 (4)	C6—C7	1.374 (5)
N3—C2	1.354 (4)	C1—H1	0.9300
N4—C3	1.320 (4)	C2—H2	0.9300
N4—C7	1.333 (4)	C4—H4	0.9300
N5—C9	1.366 (4)	C5—H5	0.9300
N5—C8	1.357 (4)	C6—H6	0.9300
N5—C7	1.415 (4)	C8—H8	0.9300
N6—N7	1.391 (4)	C9—H9	0.9300
N6—C9	1.296 (4)	C12—C13	1.373 (5)
N7—C8	1.291 (4)	C13—C14	1.367 (4)
O4—H4A	0.8500	C14—C15	1.384 (5)
O4—H4B	0.8500	C15—C16	1.374 (5)
N8—C11	1.291 (4)	C10—H10	0.9300
N8—N9	1.393 (4)	C11—H11	0.9300
N9—C10	1.301 (4)	C13—H13	0.9300
N10—C12	1.416 (4)	C14—H14	0.9300
N10—C10	1.368 (4)	C15—H15	0.9300
N10—C11	1.351 (4)	C17—H17	0.9300
N11—C16	1.323 (4)	C18—H18	0.9300
			
H1A—O1—H1B	114.00	N3—C1—H1	125.00
H2A—O2—H2B	115.00	N2—C2—H2	125.00
H3A—O3—H3B	117.00	N3—C2—H2	125.00
N2—N1—C1	107.2 (3)	C3—C4—H4	121.00
N1—N2—C2	106.8 (3)	C5—C4—H4	121.00
C1—N3—C2	104.7 (2)	C4—C5—H5	120.00
C1—N3—C3	128.3 (2)	C6—C5—H5	120.00
C2—N3—C3	127.0 (2)	C5—C6—H6	121.00
C3—N4—C7	116.5 (2)	C7—C6—H6	121.00
C8—N5—C9	104.1 (2)	N5—C8—H8	124.00
C7—N5—C8	128.2 (3)	N7—C8—H8	124.00
C7—N5—C9	127.7 (3)	N6—C9—H9	125.00
N7—N6—C9	106.9 (3)	N5—C9—H9	125.00
N6—N7—C8	107.1 (3)	N9—C10—N10	110.7 (3)
H4A—O4—H4B	115.00	N8—C11—N10	111.6 (3)
N9—N8—C11	106.9 (3)	N10—C12—C13	121.4 (3)
N8—N9—C10	106.8 (3)	N11—C12—C13	124.5 (3)
C10—N10—C12	127.6 (3)	N10—C12—N11	114.1 (3)
C10—N10—C11	104.1 (2)	C12—C13—C14	117.2 (3)
C11—N10—C12	128.3 (3)	C13—C14—C15	120.5 (3)
C12—N11—C16	116.5 (3)	C14—C15—C16	116.7 (3)
C17—N12—C18	104.4 (2)	N11—C16—C15	124.7 (3)
C16—N12—C17	128.1 (2)	N12—C16—C15	121.1 (2)
C16—N12—C18	127.4 (2)	N11—C16—N12	114.2 (2)
N14—N13—C17	107.3 (3)	N12—C17—N13	110.6 (3)
N13—N14—C18	106.3 (3)	N12—C18—N14	111.3 (3)
N1—C1—N3	110.5 (3)	N10—C10—H10	125.00
N2—C2—N3	110.9 (3)	N9—C10—H10	125.00
N3—C3—C4	121.9 (3)	N10—C11—H11	124.00
N4—C3—C4	124.4 (3)	N8—C11—H11	124.00
N3—C3—N4	113.7 (2)	C14—C13—H13	121.00
C3—C4—C5	117.5 (3)	C12—C13—H13	121.00
C4—C5—C6	120.0 (3)	C13—C14—H14	120.00
C5—C6—C7	117.1 (3)	C15—C14—H14	120.00
N4—C7—N5	113.9 (2)	C14—C15—H15	122.00
N5—C7—C6	121.5 (3)	C16—C15—H15	122.00
N4—C7—C6	124.6 (3)	N13—C17—H17	125.00
N5—C8—N7	111.2 (3)	N12—C17—H17	125.00
N5—C9—N6	110.7 (3)	N12—C18—H18	124.00
N1—C1—H1	125.00	N14—C18—H18	124.00
			
C1—N1—N2—C2	−0.3 (4)	C12—N10—C10—N9	−178.3 (3)
N2—N1—C1—N3	0.0 (4)	C11—N10—C12—N11	178.5 (3)
N1—N2—C2—N3	0.4 (4)	C11—N10—C12—C13	−1.4 (5)
C2—N3—C1—N1	0.2 (4)	C10—N10—C12—N11	−3.5 (5)
C3—N3—C1—N1	−176.3 (3)	C10—N10—C12—C13	176.5 (3)
C1—N3—C2—N2	−0.3 (4)	C16—N11—C12—N10	−179.6 (3)
C3—N3—C2—N2	176.2 (3)	C12—N11—C16—C15	0.5 (5)
C1—N3—C3—N4	170.9 (3)	C16—N11—C12—C13	0.4 (5)
C1—N3—C3—C4	−7.3 (5)	C12—N11—C16—N12	−178.6 (3)
C2—N3—C3—N4	−4.9 (5)	C17—N12—C16—C15	−3.5 (5)
C2—N3—C3—C4	176.9 (3)	C18—N12—C16—N11	−2.4 (5)
C7—N4—C3—N3	−178.4 (3)	C18—N12—C16—C15	178.5 (3)
C7—N4—C3—C4	−0.2 (5)	C16—N12—C17—N13	−178.6 (3)
C3—N4—C7—N5	−178.4 (3)	C18—N12—C17—N13	−0.2 (4)
C3—N4—C7—C6	0.8 (5)	C16—N12—C18—N14	178.5 (3)
C8—N5—C7—N4	−173.2 (3)	C17—N12—C16—N11	175.6 (3)
C8—N5—C7—C6	7.6 (5)	C17—N12—C18—N14	0.1 (4)
C9—N5—C7—N4	6.3 (5)	C17—N13—N14—C18	−0.2 (4)
C9—N5—C7—C6	−172.9 (3)	N14—N13—C17—N12	0.3 (4)
C7—N5—C8—N7	179.9 (3)	N13—N14—C18—N12	0.1 (4)
C9—N5—C8—N7	0.3 (4)	N3—C3—C4—C5	177.4 (3)
C7—N5—C9—N6	−180.0 (3)	N4—C3—C4—C5	−0.7 (5)
C8—N5—C9—N6	−0.5 (4)	C3—C4—C5—C6	1.0 (5)
C9—N6—N7—C8	−0.2 (4)	C4—C5—C6—C7	−0.5 (5)
N7—N6—C9—N5	0.4 (4)	C5—C6—C7—N4	−0.4 (5)
N6—N7—C8—N5	−0.1 (4)	C5—C6—C7—N5	178.6 (3)
N9—N8—C11—N10	0.5 (4)	N10—C12—C13—C14	179.4 (3)
C11—N8—N9—C10	−0.5 (4)	N11—C12—C13—C14	−0.6 (5)
N8—N9—C10—N10	0.3 (4)	C12—C13—C14—C15	0.0 (5)
C11—N10—C10—N9	0.0 (4)	C13—C14—C15—C16	0.7 (5)
C10—N10—C11—N8	−0.3 (4)	C14—C15—C16—N11	−1.0 (5)
C12—N10—C11—N8	178.0 (3)	C14—C15—C16—N12	178.0 (3)

### Hydrogen-bond geometry (Å, °)

**Table:**

*D*—H···*A*	*D*—H	H···*A*	*D*···*A*	*D*—H···*A*
O1—H1A···N1^i^	0.85	1.98	2.814 (5)	165
O1—H1B···N7^ii^	0.85	2.07	2.904 (5)	168
O2—H2A···O4	0.85	1.87	2.709 (5)	169
O2—H2B···O1	0.85	2.00	2.850 (6)	174
O3—H3A···O1^iii^	0.85	2.05	2.888 (5)	169
O3—H3B···O2	0.85	2.07	2.916 (6)	170
O4—H4A···N13^iv^	0.85	1.98	2.822 (5)	170
O4—H4B···N8^v^	0.85	2.06	2.873 (5)	159

Symmetry codes: (i) −*x*+1, *y*−1/2, −*z*+1; (ii) *x*, *y*, *z*+1; (iii) *x*+1, *y*, *z*; (iv) −*x*, *y*−1/2, −*z*; (v) *x*−1, *y*, *z*−1.
